# A qualitative study on gender barriers to eye care access in Cambodia

**DOI:** 10.1186/s12886-018-0890-3

**Published:** 2018-08-29

**Authors:** Camille Neyhouser, Ingrid Quinn, Tessa Hillgrove, Renee Chan, Chhorvann Chhea, Seang Peou, Pol Sambath

**Affiliations:** 1 The Fred Hollows Foundation, Level 2, 61 Dunning Avenue, Rosebery, Sydney, NSW 2018 Australia; 2 School of Public Health and Community Medicine, Faculty of Medicine, University of New South Wales, Kensington, NSW 2052 Australia; 3 Siem Reap, Cambodia; 4 Phnom Penh, Cambodia; 5 The Fred Hollows Foundation Cambodia, Phnom Penh, 12301 Cambodia

**Keywords:** Barriers, Cambodia, Eye care, Gender role

## Abstract

**Background:**

The Fred Hollows Foundation (FHF) Cambodia recently partnered with the Ministry of Women’s Affairs (MoWA) and National Program for Eye Health (NPEH, part of the Ministry of Health) to establish the *Gender Equality in Eye Health Project*. As part of this project, a qualitative study was carried out to identify barriers affecting women’s access to eye health in Cambodia.

**Methods:**

A cross-sectional qualitative study was conducted in four provinces in both urban and rural locations between May and June 2015. Purposive sampling was used to identify respondents from a range of age groups, geographical locations, and experiences to explore different perceptions regarding access barriers to eye health care. Thirteen women experiencing eye problems (age range 45–84 years; mean age 63 years) and 25 eye health professionals took part in in-depth interviews. Eleven focus groups discussions were held with 69 participants (50 women, 19 married men) to capture the views and experiences of both younger and older women, as well as household decision makers’ perspectives.

**Results:**

Gender-based differences in decision-making, access and control over resources and women’s social status all contributed to impeding women’s access to eye health services. Women relied predominantly on informal sources of information about health, and these channels might be utilised to address barriers to information and access. Disparities in perceived costs of eye health treatment were evident between eye healthcare providers and users: costs were not perceived as a barrier by service providers due to health financing support for poor patients, however, many users were not aware of the availability of the scheme.

**Conclusion:**

Demand-side and supply-side elements interact to reduce women’s ability to seek eye treatment.

## Background

Women make up approximately 60% of the estimated 223.4 million people who live with avoidable blindness or visual impairment [1]. A major cause of blindness is cataract, which can be successfully treated by surgery [1]. Globally, although women bear the greater burden of cataract, they are less likely to receive surgery [2]. Cambodia is no exception. A rapid assessment of avoidable blindness (RAAB) conducted in 2007 estimated the prevalence of blindness to be higher among women over 50 years at 3.4%, compared to 2% of the male population of the same age [3]. Only 31% of women requiring surgery were receiving it, compared to 40% of men [3].

Previous studies conducted in Cambodia on eye health promotion and service provision have suggested potential barriers to uptake for women. These have included: cost [3, 4]; fear of surgery and poor outcomes [3–5]; fear of hospitals [5]; poor awareness of eye health issues and treatment [3–5]; and a lack of social support [3, 4, 6]. The majority of these studies focussed on individual determinants of eye health behaviours. However, factors promoting health-seeking behaviours are not rooted solely within the individual. Women are vulnerable as a result of broader social constructs and in Cambodia, it is not well understood how these constructs influence access to eye health care. In addition, these previous studies did not explore provider-side barriers to understand aspects inherent to the health system that hinder service uptake, nor the perspectives of men on women’s eye health.

The purpose of this study was to inform projects aiming to increase women’s access to eye health services for The Fred Hollows Foundation (FHF), which is a secular non-profit non-government organisation (NGO) with a vision for a world where no one is needlessly blind [7]. FHF currently has programs running in more than 25 countries and has been operating in Cambodia since 1998 [7]. Therefore this study aimed to broaden our understanding of the socio-ecological determinants for women accessing eye health care in Cambodia with a view to design an evidence-based intervention to ensure delivery of gender equitable eye services in Cambodia in collaboration with the Cambodian Ministry of Women’s Affairs.

The research objectives were:

1. To identify demand-side (consumer) barriers for women accessing eye health care
2. To identify supply-side (provider) barriers for women accessing eye health care
3. To identify barriers to accessing eye health care for a vulnerable sub-group of women

This paper aims to identify barriers affecting women’s access to eye health in Cambodia in order to inform the development of an evidence-based intervention addressing these barriers. Specifically, this paper describes demand-side barriers, supply-side barriers and intersecting barriers affecting a vulnerable sub-group of women.

## Methods

### Study area

**Fig. 1:**
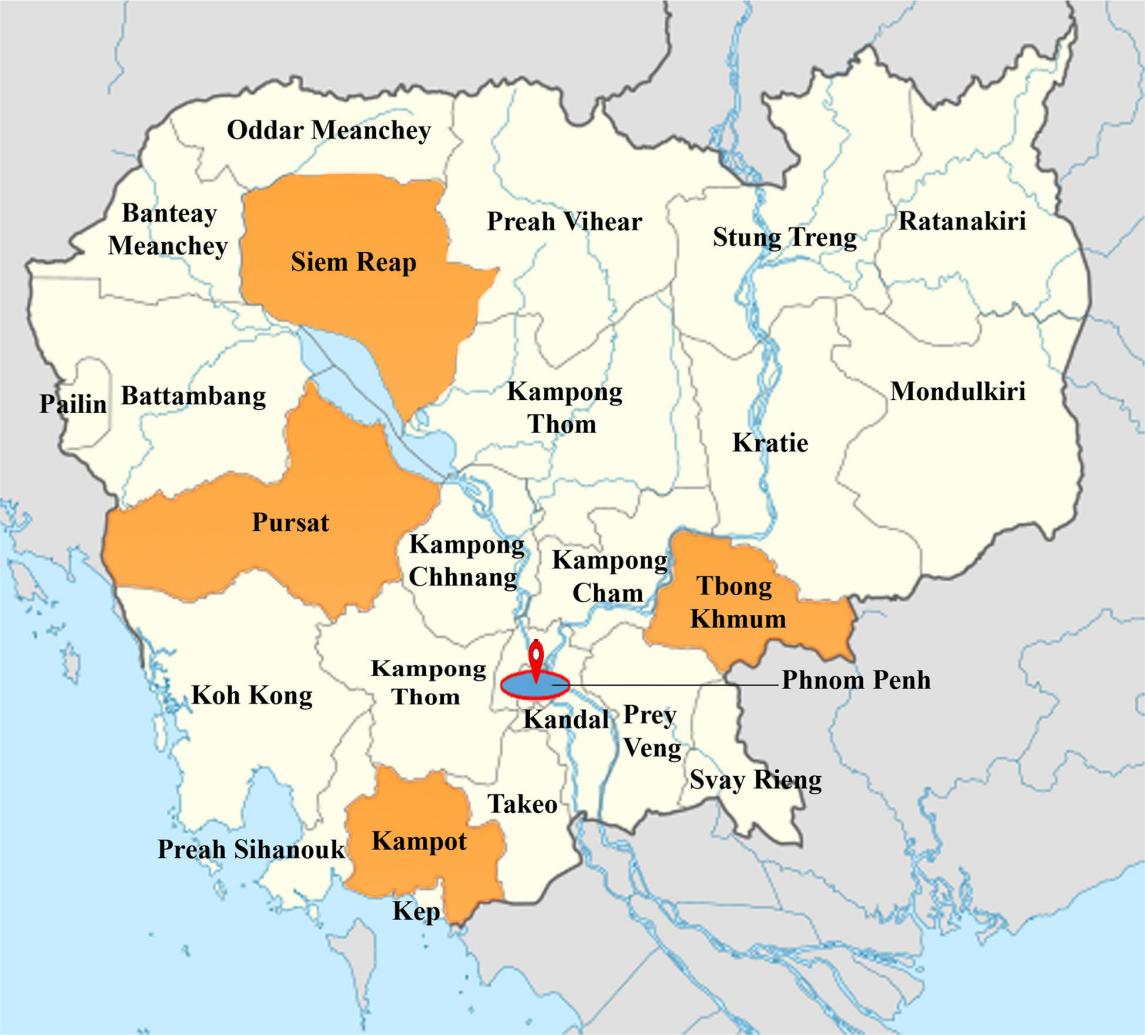
Map of Cambodia showing the study areas: Siem Reap, Kampot, Pursat and Tbong Khmum provinces. Legend  Study areas. Source: Adapted from: Location map of Cambodia by NordNordWest available at https://commons.wikimedia.org/wiki/File:Cambodia_adm_location_map.svg. Licence: Creative Commons by-sa-3.0 de

The study was conducted in urban and rural locations in four provinces of Cambodia: Siem Reap, Kampot, Pursat and Tbong Khmum. Provinces were pre-selected based on purposive criteria including poverty levels and availability of eye health programs/services. Exact study locations were selected by the Cambodian researcher (CC) in consultation with FHF Cambodia. The local eye health researcher has an in-depth knowledge of the Cambodia context since he belongs to the National Institute for Public Health (NIPH). Siem Reap was also selected, as it is a hub for FHF Cambodia, which has many partnerships in the area. The selection was not intended to be representative for the whole country, but rather to provide insights into the barriers to eye health care access for women in a diverse range of settings in Cambodia. A map of Cambodia showing the study areas is depicted in Fig. 1.

### Study design and sampling

This qualitative study engaged a gender equity approach which is concerned with the role of gender relations in the production of vulnerability to ill-health or disadvantage within health care systems [8]. This study utilised focus-group discussions (FGDs), in-depth interviews (IDIs), and key informant interviews (KIIs) to collect information on perceptions and experiences regarding access barriers to eye health care amongst different subgroups of women, men and service providers in Cambodia.

The FGDs were conducted at the community level. A non-probability purposive sampling approach was used, with participants selected according to their demographic and social characteristics, including gender, level of education, socio-economic status (as measured by income) and geographic location (urban/rural) in each study location. Groups identified for inclusion are shown in Table 1.

**Table 1:** Demographic characteristics of focus-group discussion (FGD) participants

	Marital status	Age group	No. of participants
1.	Urban women	<50 years	7
2.	Urban women	>50 years	12
3.	Rural women	<50 years	11
4.	Rural women	>50 years	20
	Sub-total women in FGDs		50
5.	Urban married men	<50 years	9
6.	Rural married men	<50 years	5
7.	Rural married men	>50 years	5
	Sub-total men in FGDs		19
	Total FGD participants		69

IDIs were conducted to collect information on perceptions and experiences of individual women experiencing eye health problems. Participants were selected through a combination of convenience and purposive sampling. IDIs were conducted with patients seeking eye treatment on a selected day at each of the four provincial referral hospitals, identified by Eye Unit personnel. Participants with an existing eye problem but who had not sought eye treatment were identified by Village Health Volunteers (VHVs), who were familiar with the health needs of local community members. A list of IDI participants’ demographic characteristics is shown in Table 2.

**Table 2:** Demographic characteristics of in-depth interview (IDI) participants

No.	Age	Education	Place Interviewed
Women with eye problems who sought eye treatment			
1	60	Illiterate	Siem Reap Provincial Hospital, Eye Hospital
2	69	Illiterate	Siem Reap Provincial Hospital, Eye Hospital
3	63	Informal education	Kampot Referral Hospital, Eye Unit
4	66	Grade 4	Tbong Khmum Referral Hospital, Eye Unit
5	84	Illiterate	Pursat Referral Hospital, Eye Unit
6	50	Illiterate	Pursat Referral Hospital, Eye Unit
Women with eye problems who did not seek eye treatment			
7	68	Grade 2	Popel Health Centre
8	60	Illiterate	Popel Health Centre
9	58	Grade 6	Tany Health Centre
10	45	Grade 6	Tany Health Centre
11	80	Illiterate	Tbong Khmum Referral Hospital
12	58	Illiterate	Chi Peang Health Centre
13	59	Grade 1	Chi Peang Health Centre
	Total IDI participants: 13		

KIIs were conducted to gain an understanding of health sector service provider perceptions regarding barriers to accessing eye health care for women. Key informants were selected for interviews through purposive sampling at the provincial and district levels based on their knowledge of eye health care management/operations and of eye health service delivery. A list of key informants’ positions and gender is shown in Table 3.

**Table 3:** List of key informant interview (KII) participants per function and gender

Key informant interview (KII) participant’s role	Male	Female
Coordinator, National Program for Eye Health (NPEH)	1	0
Country Director, Fred Hollows Foundation Cambodia	1	0
Director or Deputy Directors of Provincial Health Department	4	0
Referral Hospital Eye Health Providers	3	0
Health Centre Facility Managers	4	0
Commune Committee for Women and Children (CCWC) Representatives	0	4
Village Health Volunteers	3	5
Total KII participants per gender	16	9

### Data collection

Data collection took place over 3 weeks between May 2015 and June 2015. The international researcher (IQ) conducted the FGDs, IDIs and KIIs with the assistance of a national research assistant/translator (BC). All were conducted using simultaneous Khmer-English translation with the exception of the KIIs with the eye health service provider in Siem Reap, FHF Cambodia Country Manager and National Program for Eye Health (NPEH) Coordinator, all conducted in English. Three semi-structured interview guides were developed in English, reviewed by the local eye health consultant, translated, pilot tested and used to conduct the FGDs, IDIs and KIIs. IDIs were conducted in provincial eye hospitals, in eye units of district referral hospitals or in district health centres. KIIs took place at the workplace location of each interviewed participant. FGDs were conducted at district health centres in each province with the meeting area enclosed for privacy wherever possible. Each FGD was approximately 2 h long while IDIs and KIIs lasted approximately 1 h.

### Data analysis

Field audio recordings in Khmer were transcribed to English by BC, while audio recordings from interviews in English were transcribed verbatim by IQ. IQ was responsible for undertaking data analysis with support from BC. The data were analysed by using a systematic manual text-analysis procedure. First, a preliminary review of the raw data was conducted to reduce and generate specific thematic categories and codes. The codes included key words that represented topics conveyed in the transcripts. More specific descriptive sub-codes were assigned to data grouped under these broad categories. Searches were then conducted for each sub-code to bring together text from all FGDs and individual interviews related to each theme. As part of this step, tables were created to list main themes, subthemes, and all quotes related to each subtheme, to determine patterns in the data according to source and summarize perceptions on barriers to access to eye health service use.

## Results

### Characteristics of the study population

A total of 108 participants (72 women, 36 men) were interviewed. Eleven FGDs were held with a total of 69 participants (50 women,19 married men). The women for the FGDs were selected to provide a perspective of female eye health consumers. Men were selected to provide a perspective of married men as heads of household. In addition, 13 face-to-face IDIs were conducted with women whose ages ranged between 45 and 84 years (mean age 63 years) experiencing eye problems to provide more insight into barriers for women in accessing eye health care. Out of these 13 women, six were currently receiving eye treatment at one of the provincial referral hospitals in Siem Reap, Tbong Khmum, Kampot or Pursat. Five were from rural areas and one woman was from an urban area. Seven women who were all from a rural area had eye problems but did not seek eye treatment. Most IDI participants were illiterate or had low education levels. Furthermore, KIIs were conducted with 25 participants (9 women, 16 men) who were representatives of relevant line ministries and government departments, professional eye health care providers and health facilitators at commune level. The purpose of the KIIs was to explore barriers to eye health care access for women from a supply side perspective.

### Demand-side barriers to accessing eye health care for women

In IDIs and FGDs, study participants were asked about the barriers they encountered in accessing eye health care services. Four main themes emerged: socio-cultural factors, access and control over resources, organisational/institutional factors, and economic factors.

*Socio-cultural factors* are related to socio-cultural realities in Cambodia. They are the result of larger power differentials embedded within the community leading to patriarchal attitudes and deep-rooted gender stereotypes. Men’s health is prioritised due to the societal perception that the potential benefits to both the household and the community is higher for men than women. Because women have less agency over their own health than men and are traditionally the primary caretakers, they reported often having to negotiate with their husbands and/or family to organise their childcare and household duties in order to access health care:

*If I had eye surgery, I would stay in bed and who will look after me? And who will look after my grandchildren? I decided not to go.* (Female participant, 60 years old, rural area)

Many female participants also displayed a number of beliefs about eye health. Most deemed eye problems, including visual impairment, as being ‘not serious’ enough to seek treatment, unless experiencing pain. Further, many female respondents believed vision impairment to be an inevitable but natural consequence of aging and that eye treatment interventions require surgery and a prolonged recovery period. A fear of surgery further served as a deterrent to seeking eye treatment.

*Access and control over resources* relates to household resources and access to accurate health-related information. Because of women’s lack of access over household resources, attitudes of male heads of household were important in either supporting or discouraging women from seeking eye health care, including financially:

*I would not go if my husband does not give me the money.* (Female participant, 55 years old, urban area)

For elderly women undergoing eye treatment, the cost of treatment is often shared between adult children and decision-making is a collective process requiring consensus. Adult children are therefore an important source of information, as well as financial and psychological support for women:

*I was encouraged by my sons and daughters to go to the hospital for treatment.* (Female participant, 60 years old, urban area)

In spite of the availability of a wide range of information sources in the community, it was found that the status of women prevented them from accessing accurate information and effective communications:

*Men have more opportunities [to access information] than women. For example, men can go out to the coffee shop, or join the meeting or public discussion…but for women, few go out, they just stay at home.* (Director, Referral Hospital, male)

Participant perceptions of eye health care were largely determined by informal sources of information such as word of mouth and treatment outcomes observed in their direct environment, rather than by information from health services. Unsurprisingly, women who decided to seek treatment were often motivated to do so by their family circle or social networks:

*I was encouraged by my sons and daughters to go to the hospital for treatment.* (Female participant, 60 years old, urban area)

However, these informal sources were often shown to be inaccurate resulting in misconceptions regarding eye treatment:

*Some people say I have to spend hundreds of dollars for the treatment, particularly surgery.* (Female participant, 68 years old, rural area)

In terms of *institutional/organisational factors*, neither female nor male participants sought eye health care at the health centre (HC), as participants perceived that eye health services at HCs were at best limited.

*I don’t come because I don’t think the health centre has the medicine that I need for eye health problems.* (Female participant, 57 years old, rural area)

However, women were less likely to be able to access eye health units of provincial referral hospitals due to costs incurred by long travel distances and cultural norms dictating that they should not travel alone. In addition, their lack of experience and familiarity with health systems and structures was seen as a significant barrier, particularly for elderly and poorly educated women. For instance, a lack of support/assistance with hospital administrative processes was reported by several participants.

*Economic constraints* posed a considerable barrier to accessing eye health care for women, both in terms of perceived direct costs (user fees/treatment costs) as well as indirect costs (transportation, opportunity costs e.g. lost income):

*I have never had any [eye] treatment, this is my first time… the problem I have is not serious and the cost of the treatment... I think I have to pay a lot of money and the transportation is also costly, so I just ignore it.* (Female participant, 68 years old, rural area)

In addition, although in Cambodia poor patients are entitled to receive free or discounted care at public facilities through equity cards, in practice they reported this was rarely the case. It was evident that participants were often unable to effectively utilize these financial support schemes due to a lack of awareness of health financing support and/or a lack of understanding of how to navigate the system to obtain financial support.

### Supply-side barriers to accessing eye health care for women

KIIs with service providers explored barriers to eye health care access for women from a supply side perspective across four dimensions: accessibility, availability, affordability and acceptability.

In this study, *accessibility of services* refers to location of services, transportation and opportunity costs including travel time. Limited availability of eye services at the HCs and long distance to the referral hospitals emerged as significant challenges for women seeking eye treatment. Eye specialists are primarily located in urban areas, which contributes to the up-front transportation costs for rural patients, particularly for women who have less access to resources and limited decision-making power regarding health care expenditure. In addition, cultural norms dictate that women should not travel alone, particularly long distances and that patients are accompanied for the duration of hospital treatment. The challenge of finding an accompanying person, particularly for elderly and visually impaired women, was reported as a barrier to access.

The success of eye outreach programmes and eye camps organised by NGOs, in collaboration with the government shows that, in the absence of such initiatives, accessibility issues disproportionately impact women. This is reflected in the increase in female patients attending provincial hospital eye units for treatment following referral, as observed at one referral hospital:

*More and more patients come here for eye treatment and the number of female patients who come to the eye hospital is higher than men.* (Eye Health Professional, Referral Hospital, male)

Limited *availability* of eye health human resources was acknowledged by service providers as contributing to extending patient waiting times. For women in urban higher income groups, waiting times were reported as an important factor in decision-making. Similarly, FGDs showed that waiting times were a barrier for rural women seeking eye health care, tied to opportunity costs including travel time and lost income. In addition, supply-side respondents recognised that amongst female eye health consumers, availability of information required to make informed decisions regarding eye health was limited. At community level, VHVs recognized that whilst women in rural areas often relied on health messages delivered by VHVs, reaching vulnerable sub-groups, especially elderly women was a continued challenge:

*Currently, people trust VHV’s information. [However] If we call people for meeting, very few people come… Elderly women who stay at home are unable to access health information.* (VHV, female)

A number of providers recognized that for patients unfamiliar with the health system, particularly visually impaired patients from remote areas, the modern hospital environment may be perceived as complex and intimidating. This may act as a deterrent to eye health utilization, particularly amongst poor rural women.

In terms of *affordability*, patient treatment costs were not perceived as a barrier to access by the majority of service providers due to the range of health financing support mechanisms in place for poor patients (e.g. user fee exemptions for equity card holders). In reality, however, these mechanisms have not resulted in the removal of cost barriers for eye health consumers, as described in the paragraph on economic constraints of the Demand-side section. One provider recognized the broad financial implications of accessing eye care services for patients, including indirect costs:

*Yes, of course it is a burden for the [poor] patients...When they come and stay at the hospital, they have to pay… for treatment fees and their living expenses… they may need their relatives such as sons or daughters to come along and look after them. So those relatives cannot work or earn any money for the family. That really makes the situation worse.* (Deputy Director, Provincial Health Department, male)

Strong traditional beliefs as well as fear of surgery and negative treatment outcomes were cited by service providers as *acceptability barriers* for women:

*They are afraid of the result of the operation. Some believe that their eyes may get worse after the surgery... that surgery might be painful, and… that the result may be unsatisfactory… This may deter women from seeking treatment.* (Commune Committee for Women and Children Representative, female)

They also recognised that the effectiveness of the referral system is limited and its shortcomings disproportionately impacted women.

*We need to strengthen referral system and make consumers/patients aware of services at the health centre.* (Eye Health Professional, Referral Hospital, male)

Health providers acknowledged that women’s multiple roles and responsibilities acted as barriers and that the interaction of gender-specific barriers had an important influence on their ability to seek eye health services, particularly elderly women.

*I think the problems is not having caretakers; there is no one to replace them, to take over their responsibilities… grandparents that have to look after the household and prepare food for children. If they have eye surgery, they have to take a rest and somebody else has to do the work…* (Eye Health Professional, Referral Hospital, male)

A table summarising the study results in terms of demand- and provider-side barriers is presented in Table 4.

**Table 4:** Summary of barriers to accessing eye health care for women in Cambodia from a demand- and provider-side perspective

Demand-side barriers to accessing eye health care for women in Cambodia				Provider-side barriers to accessing eye health care for women in Cambodia			
Socio-cultural factors	Access to and control over resources	Institutional & organisational factors	Economic factors	Accessibility	Availability	Affordability	Acceptability
Status of women in Cambodian society Women as primary caretakers Beliefs about eye health Women’s limited agency in healthcare decision-making	Lack of control over household resources Limited access to accurate eye health information	Poor perceived quality of eye health care from service user perspective Limited eye health services at health centre level Lack of experience and familiarity with health systems and structures esp. referral Long travel distances to eye health services	Direct costs (user fees/treatment costs) Indirect costs (transportation, opportunity costs e.g. lost income) Unable to use financial schemes available for poor patients	Limited availability of eye services at the HCs Long distances to the referral hospitals Cultural norms dictate that women should not travel alone Difficult to find an accompanying person	Limited availability of eye health human resources Long waiting times for patients Limited availability of information required to make informed decisions on eye health Complexity of modern hospital environment	Patient treatment costs not perceived as a barrier to access by the majority of service providers	Strong traditional beliefs Fear of surgery and negative treatment outcomes Shortcomings in the referral system Women’s multiple roles and responsibilities

### Most vulnerable subgroups

Elderly women from lower socio-economic backgrounds living in remote rural areas were the most vulnerable group to demand-side barriers to accessing eye health. They were more likely to have lower levels of education and be less mobile. Therefore, they were more likely to experience a vision impairment compared to their urban and younger peers or married men. Multiple barriers intersect for them.

Socio-cultural factors were found to have more impact amongst those sub-groups of the population with the strongest traditional beliefs:

*Some older people…said that it is due to aging and it [eyesight] may get better... when your children are grown.* (Female participant, 46 years old, rural area)

Widowed and elderly female heads of households were even less likely to have access to information, be aware of treatment options or to seek treatment. As a result of visual impairment, their social networks were more likely weaker than other subgroups (e.g. urban married women). Due to their eye health condition, they were at a higher risk of being dependent on others at the same time as socially isolated:

*I could not do anything, even pressing the phone buttons. I cannot do anything besides staying at home and cleaning the house.* (Female participant, 60 years old, urban area)

## Discussion

This qualitative study demonstrated how women encounter barriers at various steps of their journey towards better eye health – from their individual beliefs and attitudes towards eye health to socio-cultural barriers in the household and the wider community to economic constraints and institutional barriers. Overall, a lack of access to information, fear of surgery and negative outcomes, costs of accessing eye health services and their limited availability are the most significant barriers to women’s access to eye health care services. The socio-cultural status of women means they are often not in a position to prioritise their own health. These findings are consistent with what is known about gender barriers to accessing health care services from previous research [4, 5, 7, 8].

This research shows that the demand and supply side elements interact to influence women’s ability to seek eye treatment. However, while female consumers were not aware of user fee exemptions for poor patients, and identified significant direct and indirect costs of accessing services, the vast majority of service providers did not think that costs were a barrier to access for women, citing the existence of financial support mechanisms for the most vulnerable. This is an important issue which should be addressed by policy makers and service providers. The equity card scheme currently in place is not used by those who are entitled to it, which represents a threat in terms of health financing. If poor women fail to access eye care services at the early stage of an eye health condition because of financial concerns, they might end up using the service when their eye condition has become significantly worse (e.g. when experiencing pain as reported by several women), risking a higher cost for the health financing system. Another risk is that patients may become irreversibly blind as a result of not accessing the services, compounding the disadvantage for their family, their community and the broader society.

Addressing socio-cultural barriers is an important step towards better eye health for women. Across all age groups and locations, women seek advice and approval from their social networks in weighing the advantages and disadvantages of seeking treatment and different treatment options. Women are more vulnerable to poor, inaccurate or distorted information because their main source of information is word of mouth. If social networks are strong and well informed, women may be more likely to seek eye health care. Eye health services should therefore seek to influence informal information channels, especially adult children who are an important part of the decision-making process. The socio-cultural barriers were largely mitigated by the intervention of outreach programmes for those women who were able to obtain eye treatment. This shows that if other key barriers such as cost and transport are addressed, social-cultural barriers can also be overcome.

### Strengths and limitations

The current study adds to the existing evidence because it comprehensively articulates the different barriers and identifies the most vulnerable subgroups by comparing barriers for men and women as well as for urban and rural women. In addition, it compares and contrasts the barriers identified by respondents from the demand side and supply side.

This study had some limitations. This paper documents a qualitative study. Qualitative research provides access to rich information that allows us to explore and understand complex social phenomena, and is therefore meant to yield results that are explorative rather than definitive. Although consumer-side participants were selected according to pre-defined criteria, local authorities were responsible for inviting demand-side participants to take part in the research. This may have introduced selection bias towards those familiar with the health care system. In some locations, FGDs were organised with participants other than those requested by the research team (i.e. different demographic groups). Furthermore, although every effort was made to ensure a gender-balanced representation of provider-side stakeholders, there are more men than women in healthcare management positions in Cambodia. Therefore, gender equality among KII participants could not be reached. Finally, the primary researcher does not speak Khmer. It is possible subtleties of meaning were lost in the translation process. The focus of the translation process was therefore on meaning rather than terminology.

## Conclusions

This study has identified a range of barriers to accessing eye health services for women in Cambodia and provides some insights into strategies to overcome them. Fear of surgery and gender-based differences in decision-making, access, control over resources and social status disempower Cambodian women. These demand-side barriers interact with supply-side barriers to further impede women’s ability to seek eye health services. Vulnerable women such as older rural women are more likely to rely on informal sources of information, including social networks and adult children. These channels are key in influencing women’s eye health-seeking behaviour and might be utilised to address barriers to information and access. Providing these informal sources with accurate information is an important component to improve women’s access to eye health services.

In addition, there is a disparity or inconsistency between perceptions of eye care costs between health providers and users: costs were not perceived as a barrier by service providers due to health financing support for poor patients, however, many users were not aware of the availability of the scheme. Further research is necessary to investigate the reasons for high failure rates amongst users applying for healthcare fee exemptions, as well as to inform the establishment and implementation of policies resulting in adequately reduced direct costs for vulnerable eye health consumers in Cambodia.

Findings from this research will inform the joint development through a partnership between FHF and the Cambodian Ministry of Women’s Affairs of an evidence-based intervention that will address some of the barriers identified to ensure delivery of gender equitable eye services in Cambodia.

## Abbreviations

FGD: Focus-group discussion
FHF: The Fred Hollows Foundation
HC: Health Centre
IDI: In-depth interview
KII: Key informant interview
MoH: Ministry of Health
MoWA: Ministry of Women’s Affairs
NECHR: National Ethics Committee for Health Research
NGO: Non-government organisation
NIPH: National Institute for Public Health
NPEH: National Program for Eye Health
RAAB: Rapid assessment of avoidable blindness
VHV: Village Health Volunteer
